# Protective Effect of *Spirulina platensis* Extract against Dextran-Sulfate-Sodium-Induced Ulcerative Colitis in Rats

**DOI:** 10.3390/nu11102309

**Published:** 2019-09-29

**Authors:** Mohamed A. Morsy, Sumeet Gupta, Anroop B. Nair, Katharigatta N. Venugopala, Khaled Greish, Mahmoud El-Daly

**Affiliations:** 1 Department of Pharmaceutical Sciences, College of Clinical Pharmacy, King Faisal University, Al-Ahsa 31982, Saudi Arabia; anair@kfu.edu.sa (A.B.N.); kvenugopala@kfu.edu.sa (K.N.V.); 2 Department of Pharmacology, Faculty of Medicine, Minia University, El-Minia 61511, Egypt; 3 Department of Pharmacology, M. M. College of Pharmacy, Maharishi Markandeshwar (Deemed to University), Mullana, Ambala, Haryana 133203, India; sumeetgupta25@gmail.com; 4 Department of Biotechnology and Food Technology, Durban University of Technology, Durban 4000, South Africa; 5 Department of Molecular Medicine, Princess Al-Jawhara Centre for Molecular Medicine, School of Medicine and Medical Sciences, Arabian Gulf University, Manama 329, Bahrain; khaledfg@agu.edu.bh; 6 Department of Pharmacology & Toxicology, Faculty of Pharmacy, Minia University, El-Minia 61511, Egypt; eldaly_m@mu.edu.eg

**Keywords:** *Spirulina platensis*, ulcerative colitis, dextran sulfate sodium, tumor necrosis factor-α, interleukin-6, myeloperoxidase

## Abstract

Inflammatory bowel disease is a multifactorial inflammatory condition. This study aimed to test the protective effects of *Spirulina platensis* against ulcerative colitis (UC). UC was induced in thirty-six male Wistar rats by adding dextran sulfate sodium (DSS) to their drinking water, while a control group received only drinking water. UC rats were equally-divided into six groups that received a single oral daily dose of vehicle (DSS), sulfasalazine (SSZ, 50 mg/kg/day), chloroform or the hydroalcoholic extracts of *Spirulina platensis* (100 or 200 mg/kg/day) for 15 days, and then blood and colon samples were harvested for determination of tumor necrosis factor-α (TNF-α), interleukin-6 (IL-6), erythrocyte sedimentation rate (ESR), myeloperoxidase (MPO), and histopathology. At the end of the study, compared to time-matched controls, UC rats showed increased TNF-α (1.64-fold), IL-6 (5.73-fold), ESR (3.18-fold), and MPO (1.61-fold), along with loss of body weight (24.73%) and disease activity index (1.767 ± 0.216 vs. 0 ± 0), *p* < 0.001. These effects were prevented by SSZ treatment (*p* < 0.001 vs. DSS). The hydroalcoholic extract of *Spirulina platensis* dose-dependently modulated all DSS-induced inflammatory changes. However, the chloroform extract significantly lowered only IL-6 and ESR, but not TNF-α or MPO levels. The protective effects of the hydroalcoholic extract of *Spirulina platensis* against experimental UC involved mitigation of DSS-induced inflammation.

## 1. Introduction

Inflammatory bowel disease (IBD) is an inflammatory condition of multifactorial etiology that is characterized by increased inflammation, which can further deteriorate and induce serious complications such as colon cancer [1]. The increasing incidence rates of IBD make it a global healthcare burden, especially in westernized societies [2]. IBD has two major clinical presentations; ulcerative colitis (UC) and Crohn’s disease (CD), both of which can show either acute or chronic manifestations as well as a wide range of severity [3]. Usually, CD affects any part of the gastrointestinal tract with characteristic skip lesions. On the other hand, UC affects only the rectum and the colon, with the lesions being more homogeneous and continuous [3,4].

Familial and genome-wide association studies have revealed the complex nature of IBD pathogenesis [3,5,6]. Moreover, the interplay between altered genetic, immune, and environmental factors determines both the incidence and severity of IBD. Nevertheless, inflammation remains the main feature of IBD. In spite of the complexity of the signaling pathways involved in the inflammatory pathogenesis of IBD, the emergence of tumor necrosis factor-α (TNF-α) as a common player in clinical and experimental models is not surprising [7]. In addition, many of the interleukins (ILs) including IL-6, IL-17, IL-23, and IL-26, among others, have been implicated in IBD pathogenesis and its complications [8,9,10], while IL-10 signaling through its receptors plays a protective role against IBD development [11,12,13].

One important clinical manifestation of IBD is nutritional deficiency as a result of deteriorated gastrointestinal function, which adversely affects the patient’s general health and contributes to disease-related morbidity. Moreover, diet itself might represent an important causative factor in IBD pathogenesis [14,15]. Thus, the dietary approach, via diet modification or the use of appropriate functional foods and nutraceuticals is generally accepted for the management of patients suffering from IBD [15,16,17]. Interestingly, a recent case–control study showed that subjects without gastrointestinal diagnoses had more consumption of functional foods and better adherence to a Mediterranean diet than those diagnosed with UC, CD, irritable bowel syndrome, or gastroesophageal reflux [18]. Other reports linked consumption of unhealthy foods with higher incidence of UC [14].

The clinical use of cyanobacterium *Spirulina platensis* as a functional food and a nutritional supplement for human conditions is currently on the rise [19,20,21,22,23,24,25]. A recent systematic review highlighted both the safety and the ameliorative effects of *Spirulina* supplements on the components of metabolic syndrome in humans [20]. Interestingly, the consumption of *Spirulina platensis* supplements in infants during early life has been positively associated with better motor functional development and enhanced social skills [21,22]. In addition, a commercially available preparation based on *Spirulina* extract showed immunomodulatory effects in healthy volunteers [23]. Moreover, the consumption of this functional food has been associated with decreased vascular inflammation and markers of endothelial dysfunction in hypertensive patients [24] as well as decreased levels of inflammatory cytokines such as IL-6 in obese patients, in addition to improvement of insulin sensitivity and total antioxidant capacity in such populations [25]. Additionally, carotenoids such as lycopene and zeaxanthin, which can be found as constituents of *Spirulina* along with others such as β-carotene [26,27], which is an important source of vitamin A, have been found to clinically improve IBD-related symptoms [28]. Indeed, treatment of UC patients with vitamin A itself was positively associated with increased mucosal healing and improved clinical outcomes [29].

In addition to its current use in human studies, a growing body of evidence from animal studies supports the functional roles of *Spirulina* in protection against various disease conditions. Recent research has shown that *Spirulina* preparations have neuroprotective [30], anti-ulcer [31], hepatoprotective [32], and nephroprotective [33] effects in experimental animals. These effects are believed to be mediated via antioxidant and anti-inflammatory mechanisms [34,35].

Given the promising anti-inflammatory and antioxidant effects of *Spirulina* on one hand, and its accepted use as a safe functional food on the other, this work aimed to evaluate the possible protective effects of *Spirulina platensis* extract against dextran sulfate sodium (DSS)-induced UC in rats. We hypothesized that *Spirulina platensis* would protect against UC disease development, at least in part, via modulation of the body’s inflammatory response.

## 2. Materials and Methods

### 2.1. Chemicals

*Spirulina platensis* powder was obtained from Recon Healthcare (Bangalore, India). Analytical grade acetone, hexane, and ethyl acetate and HPLC grade methanol were purchased from Merck (Darmstadt, Germany). DSS (molecular weight 40000) was purchased from Sigma Aldrich (St. Louis, MO, USA).

### 2.2. Preparation of Spirulina platensis Extract

*Spirulina platensis* powder was extracted using either a hydroalcoholic (HA) solvent mixture or chloroform. The HA extract was prepared by macerating the *Spirulina platensis* powder in a mixture of ethanol and water (60:40, v/v) at room temperature for 7 days. The extract was then filtered, and the residue was further extracted twice using the same solvent mixture and conditions (for a total of 21 days). The chloroform extract was prepared using the same method, but instead of the ethanol and water mixture, chloroform (100%) was used for the maceration process. After 21 days of maceration, the collected filtrate was concentrated in a rotary vacuum evaporator and freeze-dried to obtain the final freeze-dried HA or chloroform extract of *Spirulina platensis* powder, which was stored at 4 °C and protected from light until used.

#### 2.2.1. Sample Preparation for High-Performance Thin-Layer Chromatography (HPTLC) Analysis

The dried HA extract sample was used for HPTLC analysis according to methods described earlier [26,36]. Briefly, 100 mg extract sample was homogenized in 1 mL acetone and hexane (1:1). The suspended sample was incubated at room temperature for 1 h then centrifuged at 12,000 rpm for 20 min at 16 °C. Afterwards, 1 mL of the resultant supernatant was transferred to a fresh 1.5 mL of methanol and then the sample was dried out in a concentrator at 37 °C. Finally, the dried extract was re-suspended in 1 mL methanol.

#### 2.2.2. Analysis of Carotenoids Using HPTLC

Separation of carotenoids was performed by using a CAMAG HPTLC system coupled with Linomat IV applicator, CAMAG TLC Scanner 3, and integrated software VisionCATS-Server-PH, version 2.5.18072.1. HPTLC was performed on 0.2 mm thick precoated silica gel HPTLC plates 60*f*_254_ (10 cm × 10 cm) (Merck). Samples were applied to the plate under a flow of N_2_ gas (dosage speed was 150 nL/s and predosage volume was 0.2 μL). Sample bands 5 mm wide, 11.4 mm apart, and 10 mm above from the bottom edge, starting 8 mm from the edge of the HPTLC plate, were applied on the plate with a Linomat IV applicator. A 10 cm × 10 cm twin-trough chamber was used for the plate development; front and rear trough volumes were 5 mL. The twin-trough chamber was saturated with the mobile phase composed of acetone and ethyl acetate (9:1, v/v) for 20 min at room temperature. The solvent front was marked at 8 cm from the bottom of the plate, at which point the plate was developed. The developed plates were dried before scanning. Qualitative evaluation of the plates was performed in absorption mode at 254 nm, using a slit width of 5 × 0.2 mm at a data resolution of 100 μm/step and scanning speed of 20 mm/s. The obtained results were used for spectrum analysis in a TLC scanner using spectrum mode from 190 nm to 900 nm and spectrum speed 20 nm/s. Data resolution was 1 nm using a slit width of 5 × 0.2 mm in computerized TLC scanner 3, furnished with VisionCATS software version 2.5.18072.1.

#### 2.2.3. Sample Preparation for Gas Chromatography–Mass Spectrometry (GC-MS)

The obtained fine powder of the HA extract was extracted in methanol (100 mg/mL) and left overnight, and then centrifuged at 8000 rpm for 20 min. The resulting supernatant was transferred into a 1.5 mL tube. The 1 mL extract was dried out in a concentrator at 37 °C. Finally, dried extract was subjected to derivatization for GC-MS analysis. GC-MS derivatization was performed by adding 70 μL of *N*-methyl-*N*-(trimethylsilyl)-trifluoroacetamide at 45 °C for 30 min. The derivatized sample was centrifuged at 12,000 rpm for 20 min, and the resulting supernatant was used for GC-MS analysis.

#### 2.2.4. GC-MS Analysis

Agilent GC-MS system comprising of Agilent 7890A gas chromatograph (Agilent Technologies, Santa Clara, CA, USA) coupled with an Agilent 5975C mass detector (Agilent Technologies) was used. Derivatized sample (1 μL) was injected into GC-MS by automatic sampler (7683 B series, Agilent Technologies) with a split ratio of 1:10. Samples were separated on a fused silica capillary column DB-5ms ((5%-phenyl)-methylpolysiloxane: 30 m length, 0.25 mm internal diameter, 0.25 µm film, Agilent Technologies). The temperature program was as follows: initial temperature of 70 °C for 5 min, followed by final temperature increase to 300 °C at the ramp rate of 10 °C/min, and finally held at 300 °C for 10 min. Total run time calculated was 38 min. The inlet temperature and interface temperature were set at 280 °C. Each sample was replicated three times. Scan was started after solvent delay of 5 min with scan frequency of 4 s^−1^ (2.0 HZ).

#### 2.2.5. Metabolite Identification in GC-MS

Metabolites present in the obtained samples were identified by library matching of mass spectra of each compound (3:1 signal to noise ratio) using the NIST-17 mass spectral library (National Institute of Standards and Technology) and our in-house database, which includes several secondary metabolites, amino acids, organic acids, and sugar standards. Metabolite identity was obtained and reported only when the matching value of the mass spectrum comparison was more than 70%.

### 2.3. Animals

An animal study protocol (MMCP-IAEC/15/31) that ethically followed the Institutional Animal Ethics Committee of M. M. College of Pharmacy, Maharishi Markandeshwar, India and the Research Ethics Committee, King Faisal University, which is in accordance with the National Committee of BioEthics (NCBE), KACST, Saudi Arabia, was followed throughout this experimental study.

Forty-two male Wistar rats weighing 200–235 g were housed three per cage in a controlled laboratory environment (25 ± 1 °C and relative humidity of 60–65%). Animals were left to acclimatize for a period of one week before the start of experiments. Rats were fed ad libitum a standard commercially-available rodent chow (supplied from Krishna Khadaya Bhandhar, Yamunanagar, Haryana, India) of 25% protein, 5% fat, 40% starch, 10% sugar, 6% fibers, 8% ash, 5% minerals (a mixture of calcium phosphate, magnesium oxide, ferric citrate, manganous carbonate, chromium sulfate, zinc carbonate, cupric carbonate, potassium iodate, and sodium selenite), and 1% vitamins (retinol, cholecalciferol, α-tocopherol acetate, menadione, thiamine, riboflavin, nicotinic acid, pyridoxine, calcium pantothenate, biotin, folic acid, and cyanocobalamin).

Rats were randomly subdivided into seven experimental groups, six animals each. The first group served as a normal control, which received only drinking water throughout the experiment and the vehicle used for drug administration (distilled autoclaved water with 0.25% tween-20) once daily by oral gavage. For induction of colitis, the other six groups were allowed free access to 3% DSS in drinking water for the first 7 days, followed by every 4th day administration (on the 11th and 15th days alternating with plain drinking water) for the total period of the study (15 days), as previously described [37,38], with slight modifications. The second group served as a positive control (DSS, no treatment except the vehicle). The remaining five groups were treated with oral sulfasalazine (SSZ) 50 mg/kg body weight, low (100 mg/kg) or high (200 mg/kg) doses of either chloroform or HA extracts of *Spirulina platensis*. All treatments were dispensed in a vehicle composed of distilled autoclaved water and 0.25% v/v tween-20 and given as a single dose by oral gavage.

#### 2.3.1. Monitoring of Animal Weight and Disease Activity Index (DAI)

The animals were monitored for changes in body weight on experimental days 1, 8, and 15. The DAI was calculated by taking the average of three parameter scores—the average body weight loss percentage, stool consistency, and the presence of fecal blood—every third day, according to previously reported methods [39,40].

#### 2.3.2. Blood and Tissue Sample Collection

At the end of the experiment, animals were anesthetized with i.p. injection of 400 mg/kg (10% solution) chloral hydrate [41]. Blood was collected by cardiac puncture (a part was used to determine erythrocyte sedimentation rate [ESR]), allowed to coagulate, and centrifuged at 10,000 rpm for 10 min to obtain the sera that were stored at −80 °C until used for analysis. Colon tissue samples were immediately harvested and cleaned gently with cold phosphate-buffered saline (PBS). Sections of the colon tissues were fixed overnight in 10% formalin in PBS solution, and were used for histopathological study. Other pieces of tissues were used for scraping of the mucosal layer for determination of mucosal myeloperoxidase (MPO) activity.

#### 2.3.3. Measurement of Cytokine Levels

Enzyme-linked immunosorbent assay (ELISA) colorimetric kits (Sigma Aldrich) were used for the measurement of serum concentrations of the inflammatory cytokines TNF-α and IL-6 according to the manufacturer’s instructions.

#### 2.3.4. Measurement of MPO Activity

MPO activity was determined (MPO ELISA kit; Sigma Aldrich) as a marker of neutrophil infiltration into the colon tissue. Briefly, mucosae were homogenized in 0.5% hexadecyltrimethylammonium bromide dissolved in PBS (pH 6.0). Supernatants were collected after tissue homogenization and centrifugation at 13,000 rpm at 4 °C for 5 min. Supernatants were mixed with the supplied buffer supplemented with 1% H_2_O_2_ and *o*-dianisidine dihydrochloride solution. Optical density of the developed color was recorded for 1 min at 30 s intervals on a microplate reader at 450 nm, as previously described [42]. Tissue protein concentration in the samples was measured with a colorimetric protein assay kit using the bicinchoninic acid method [43].

### 2.4. Statistical Analysis

Data represent the mean ± SD. Statistical analyses were carried out using Graphpad Prism. The normality of data was checked using the Kolmogorov–Smirnov test. Comparisons among groups were made with one-way or two-way ANOVA followed by Tukey’s multiple comparisons test after passing the normality test. Differences between test groups were considered significant when values of *p* were less than 0.05.

## 3. Results

### 3.1. Active Ingredients and Metabolite Profile in the Spirulina platensis Extract

**Figure 1:**
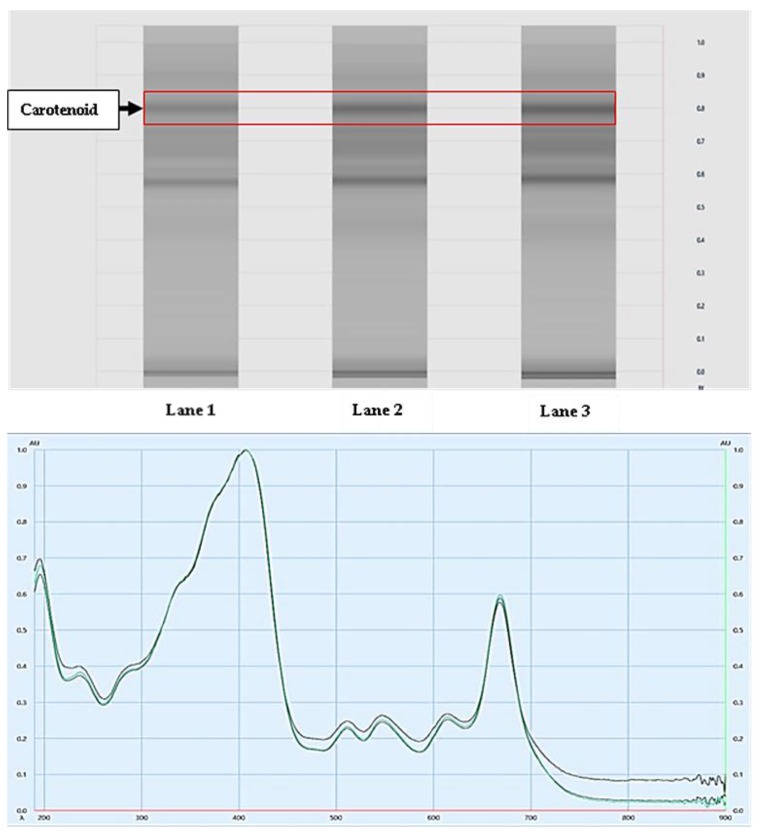
Results of high-performance thin-layer chromatography (HPTLC) analysis of *Spirulina platensis* hydroalcoholic extract. **Upper panel**: Fingerprint profile of the developed plate showing the band for carotenoids. **Lower panel**: UV spectra of the isolated carotenoids. The green line represents the spectrum of a standard carotenoid sample (β-carotene), while the black lines represent the spectra of samples recovered from the HPTLC bands (red rectangle in the upper panel).

**Figure 2:**
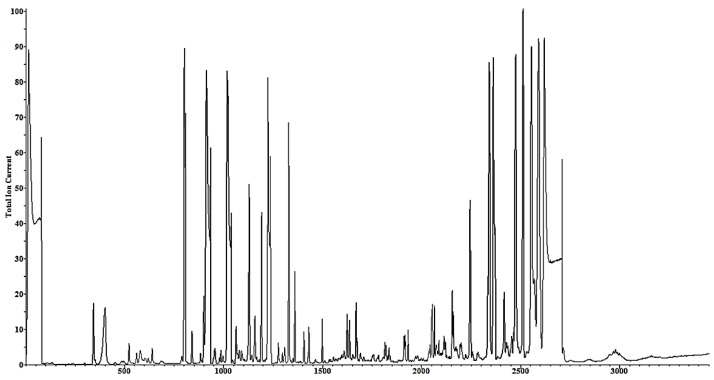
Chromatograph of the given sample run in the gas chromatography–mass spectrometry.

The HPTLC analysis of the HA extract samples was carried out in triplicates of increasing volumes of 5 µL, 8 µL, and 10 µL in Lanes 1, 2, and 3, respectively. The R_f_ values were 0.807, 0.804, 0.806 mm in the three lanes (Lane 1–3 upper panel, Figure 1), respectively. The nature of the isolated compounds as carotenoids was confirmed when samples from these bands (Figure 1, upper panel) were recovered and showed identical UV spectra when compared to standard sample β-carotene (a known standard carotenoid; Figure 1, lower panel). This experiment confirmed that the HA extract of *Spirulina platensis* contained carotenoids. On the other hand, GC-MS analysis of the sample resulted in the isolation of 24 metabolites with small molecular weight, which were identified by matching the mass spectrum of each compound using the NIST-17 mass spectral library, as shown in Figure 2 and Table 1 and Table 2.

### 3.2. Effect of DSS and Various Treatments on Animal Body Weight and DAI Scores

The data in Table 3 illustrate the effect of UC induction on the body weight of the rats. During the study period, the animals of the DSS-induced UC group showed significant reductions in body weight at Day 8 (198.50 ± 7.69) and Day 15 (174.00 ± 5.71) compared to Day 1 (220.33 ± 8.50). In addition, these animals showed dramatic weight reductions compared to the normal control group at the same time points. Similarly, animals that received the low dose (100 mg/kg) of the chloroform extract of *Spirulina platensis* demonstrated body weights comparable to those in the DSS UC group on the 8th day of treatment, but not on the 15th day, where they showed significant improvements (*p* < 0.001). However, these animals suffered significant body weight losses (*p* < 0.05) when compared to their own condition at Day 1. On the other hand, the animals in the high dose chloroform extract (200 mg/kg) group showed highly significant (*p* < 0.001) improvements in body weights compared to the DSS UC animals (positive control group). It is noteworthy that the high dose chloroform extract animals displayed lower, but non-significant, body weights in comparison to Day 1. On the other hand, animals that received either dose of the HA extract of *Spirulina platensis* were characterized by dose-dependent protection against the DSS-induced loss in body weight compared to the positive control group alone (DSS). Unlike the DSS group, the SSZ-treated animals showed non-significantly lowered body weights in comparison to Day 1, but, nonetheless, these animals had significantly higher body weights than the DSS animals on Day 15.

**Figure 3:**
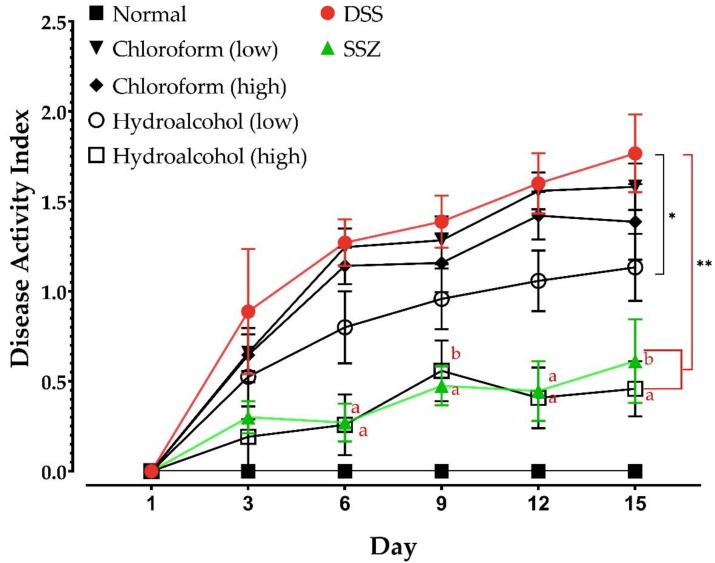
Disease activity index (DAI). DAI in all groups was calculated according to the formula: DAI = (combined score of weight loss, stool consistency, and bleeding)/3. Data (mean ± SD, n = 6) were analyzed by two-way ANOVA followed by Tukey’s multiple comparisons test. * *p* < 0.01 compared to dextran sulfate sodium (DSS) alone at all days except Days 1 and 3 (ns), ** *p* < 0.0001 comparison between the specified groups and DSS alone at all of the studied time points except at Day 1 (ns) and Day 3 (*p* < 0.05 between DSS and the high dose hydroalcoholic extract alone). a: *p* < 0.01 and b: *p* < 0.05 compared to the low dose hydroalcoholic extract. ns: not significant.

DAI was calculated in all groups on the first and third days of disease induction and then every third day throughout the experimental study. The control normal animals displayed no signs of disease (Figure 3). The DSS group, however, showed time-dependent increases in DAI, indicative of disease progression. The results in Figure 3 show that neither the low nor the high doses of the chloroform extract showed significant improvement in DAI scores compared to the untreated DSS-induced animals, although slightly lower values were obtained on the 15th day. On the other hand, the low dose of HA extract of *Spirulina platensis* group showed significant reductions (*p* < 0.01 for Days 6, 9, 12, and 15) in DAI in comparison with the DSS-induced UC animals. Interestingly, the improved DAI scores displayed by the high dose of HA extract of *Spirulina platensis* group were not significantly different from those of the SSZ-treated rats throughout the studied period, and both groups had significantly lower DAI scores than the DSS UC rats on all days starting from Day 6 (*p* < 0.0001), while only the high dose of HA of *Spirulina platensis* group showed a significant (*p* < 0.05) difference at Day 3. In addition, both groups showed significantly lower DAI than the low dose HA-treated rats, starting from Day 6 (Figure 3).

### 3.3. Effect of DSS-Induced UC and Various Treatments on Blood Inflammatory Markers

Induction of UC by oral administration of DSS for 15 days resulted in a significant increase in the levels of TNF-α (1.65-fold) and IL-6 (5.73-fold) in comparison with normal animals (Table 4), as measured after the study period. The increase in the levels of these inflammatory cytokines was beneficially modulated when animals were treated with either SSZ or with different doses of the *Spirulina platensis* extracts. Importantly, the increase in TNF-α level was totally prevented by either SSZ or the higher dose of the HA extract. Moreover, although the levels of IL-6 at the end of the experimental period (15 days) were significantly higher than in normal vehicle-treated control animals, all of them showed significantly lowered levels when compared to the DSS-induced UC positive control group. It is noteworthy that there was no significant differences between the higher dose of the HA extract-treated and the SSZ-treated rats with respect to the serum levels of either cytokine. These results show that the HA extracts were better in modulation of the inflammatory cytokines TNF-α and IL-6 than the chloroform-extract-treated groups.

Parallel to its effects on serum cytokines, induction of UC via oral administration of DSS markedly increased the ESR (~3-fold), as shown in Table 4. All treatment groups showed significantly lower ESR values than in the DSS-induced UC group. However, the low dose of HA extract group, along with both doses of the chloroform extract, still showed significantly higher ESR values when compared to time-matched, water-only rats. On the other hand, neither the SSZ-treated nor the high-dose-of-HA-extract-treated animals showed significantly different ESR values in comparison with the control animals. In addition, the higher dose of HA extract group showed significantly (*p* < 0.05) decreased ESR values relative to the low dose of the same extract group.

### 3.4. Effect of DSS and Various Treatments on Tissue Inflammation

Mucosal MPO activity is usually studied as a marker of polymorphonuclear leukocyte infiltration. In the current study, induction of UC by DSS resulted in a significant increase in tissue MPO activity in comparison with time-matched control animals (Table 5), an effect that paralleled the elevation of serum inflammatory cytokine levels. Treating the animals with either SSZ or the higher dose of *Spirulina platensis* HA extract markedly decreased tissue MPO activity.

**Figure 4:**
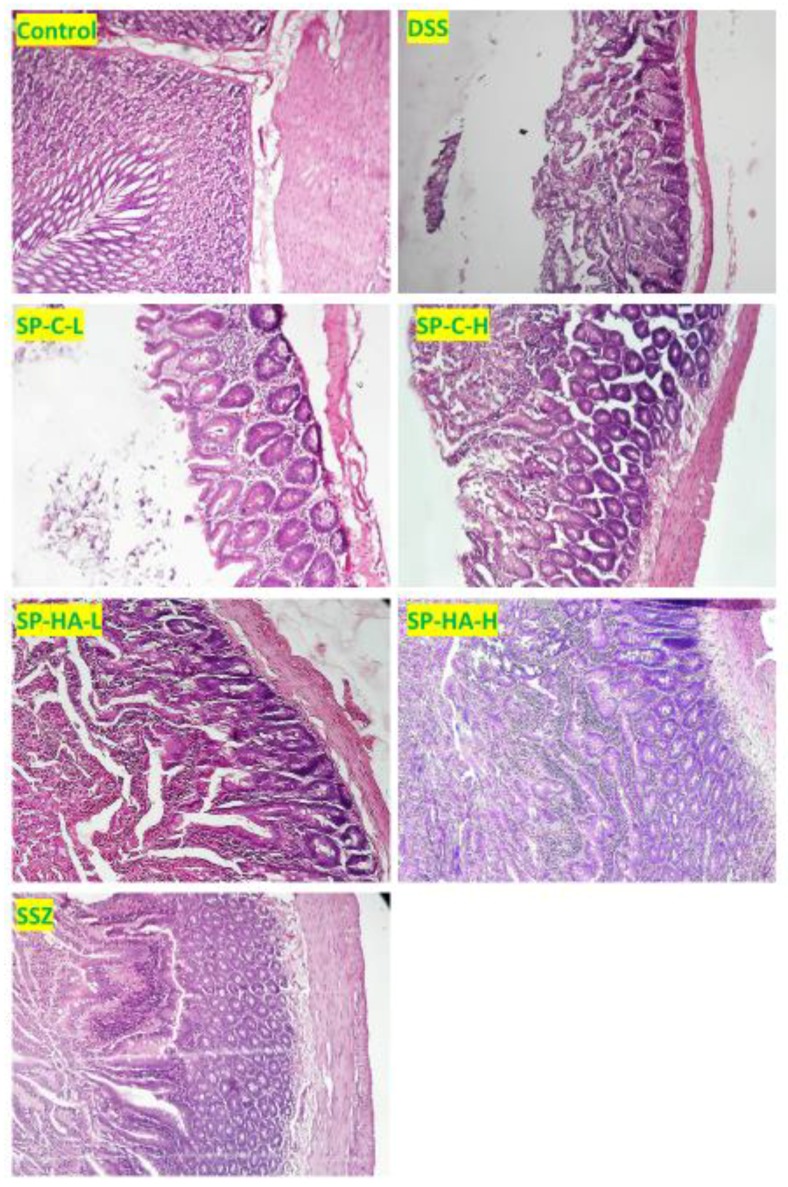
Representative photomicrographs of the colon sections after H&E staining. Control: vehicle-treated normal animals, DSS: dextran sulfate sodium positive control, SSZ: animals received DSS + sulfasalazine, SP-C-L: animals received DSS + small dose of chloroform extract of *Spirulina platensis*, SP-C-H: animals received DSS + high dose of chloroform extract of *Spirulina platensis*, SP-HA-L: animals received DSS + small dose of hydroalcoholic extract of *Spirulina platensis*, SP-HA-H: animals received DSS + high dose of hydroalcoholic extract of *Spirulina platensis*.

Histopathological examination of the DSS-induced UC group (positive control group) showed marked cellular infiltration by mononuclear cells, and several lesions of cryptic necrosis crypts were identified (Figure 4). In addition, focal ulceration, hemorrhage, and edema were also observed in the DSS-induced UC group. SSZ-treated animals showed marked reservation of cellular structures in the crypt regions, with lesser focal hemorrhage and lesser edema. Dilated blood capillaries and fewer inflammatory cells infiltration into the submucosal areas were identified in comparison with the DSS-treated group. *Spirulina-platensis*-HA-extract-treated groups showed conserved epithelial layers with massive goblet cell formation and lesser edema. In addition, less inflammatory cell infiltration in the underlying submucosa was observed than in the DSS-treated animals. On the other hand, the chloroform-extract-treated animals showed much less improvement in the regeneration of epithelium cells. Edema and crypt abscesses were noticed clearly in these groups, but there was also improvement in a few areas.

## 4. Discussion

The aim of the current study was to evaluate the protective effects of *Spirulina platensis* against experimentally-induced UC. Two dose levels of two different extracts; chloroform and HA, were evaluated in DSS-induced colitis rats, in comparison with SSZ, a standard drug usually prescribed to patients with UC [44]. The results showed that the HA extracts of *Spirulina platensis* were superior to the chloroform extracts in reducing DAI, deterioration of body weight, and local and systemic inflammation as well as histopathological damage. Moreover, the protective effects imparted by the high dose of HA extract were in many aspects comparable to those achieved by the standard SSZ treatment.

Induction of UC by oral administration of DSS is a well-established and widely accepted model of UC that mimics both the symptoms and morphological changes seen in human disease [37,45]. However, unlike in human disease, the pathogenesis of UC in this model does not necessarily require B-cell- and T-cell-mediated immune responses [3,37]. However, chronic DSS-induced UC in murine models has been reported to activate a T-cell immune response [46]. Importantly, the DSS-induced colitis model is closer to human UC than it is to CD [37,47]. The most important pathophysiological changes in human UC are inflammatory in nature [4,5,7], which are readily reproduced in this model, as shown by the results of both the current study and others [40,47]. Oral administration of DSS is well-documented to induce an acute colitis model, with significant incidence of bloody diarrhea, colon mucosal damage, and granulocyte infiltration [38,48]. In the current study, DSS-treated rats suffered marked loss in body weight, akin to the deterioration of gastrointestinal function and general animal health. The decrease in body weight in the present study was positively associated with the DAI scores. Previous research has established the negative effects of DSS on body weight as well as the general deterioration of gastrointestinal health [37,40,49].

DSS-induced UC animals in the current study manifested increased inflammatory signals, such as increased systemic levels of TNF-α and IL-6, as well as increased mucosal inflammation and ESR, a common surrogate marker of systemic inflammation [50,51]. TNF-α can be secreted by many of the cells found in the local microenvironment, including macrophages, fibroblasts, adipocytes, and dendritic and T cells [46,52]. Target cells for TNF-α mediated signaling include cells of the intestinal epithelium, the endothelium, myofibroblasts, and Paneth cells [3,53,54,55]. Activation of nuclear factor (NF)-κB and downstream signaling pathways is the central action of TNF-α receptor activation [46,53,56]. The role of NF-κB in epithelial cell death and disruption of barrier function, both hallmarks of UC, is well documented [7,56].

Mucosal inflammation in the present study was confirmed directly by assessing the pathological changes revealed by histopathological examination of colonic sections, as well as by the increased mucosal MPO activity, which is a strong indicator of tissue neutrophil infiltration [57,58]. These DSS-induced effects were confirmed by the results of previous studies showing that induction of UC was associated with colonic tissue damage characterized by enhanced cytokine levels, including TNF-α and IL-6, as well as increased immune cell infiltration [42,46].

SSZ, which is a low-cost and effective treatment for UC [44], and other inflammatory conditions [59,60] was used in this work as a standard agent to which the protective effects of *Spirulina platensis* extracts were compared. The results of this work showed that DAI scores were significantly lowered by SSZ treatment; however, the decline in body weight was only partially salvaged in SSZ-treated animals. Moreover, SSZ was associated with significantly lower inflammation markers (TNF-α, IL-6, ESR, and MPO) and tissue damage. These effects of SSZ have also been previously reported in similar experimental models [46,53,56,61,62,63].

Although the chloroform extract of *Spirulina platensis* at the low or the high doses did not completely prevent the deterioration in body weight or the elevated DAI scores, except modestly in the high-dose-treated animals, the HA extract successfully mitigated the DSS-induced body weight deterioration and improved DAI scores in a dose-dependent manner. Moreover, the protective effects achieved in the high-dose-HA-extract-treated animals were similar to those achieved by SSZ treatment. The protective effects demonstrated by *Spirulina platensis* can be attributed to its previously reported antioxidant and anti-inflammatory activities [32,34,64,65], and possibly to its supplying the animals with trace nutrients required by the body for maintenance of redox homeostasis, nutrients which can be rendered deficient as a result of disease progression [21,22,34]. Importantly, *Spirulina* is a well-known rich source of antioxidant metabolites, including carotenoids such as β-carotene and zeaxanthin as major constituents [26,66,67]. Recent clinical studies have indicated that carotenoids like β-carotene, lycopene, and zeaxanthin, as well as vitamin A, can have protective and ameliorative effects in patients with UC [28,29,67].

A few studies have addressed the question of whether oral administration of whole *Spirulina* can protect against experimental colitis, but no studies have investigated the effect of its extracts in such conditions. Coskun and his co-workers [68] showed that oral administration of whole *Spirulina* was protective against trinitrobenzenesulfonic-acid-induced colitis, which is more like human CD rather than UC [69,70]. The authors of that study attributed the protective effects of *Spirulina* to antioxidant mechanisms [68]. Moreover, a more recent study showed that oral administration of whole *Spirulina* had both antioxidant (decreased oxidative stress and enhanced endogenous antioxidant mechanisms) and anti-inflammatory (decreased inflammatory cytokine levels and neutrophil infiltration) effects in acetic-acid-induced rat colitis [71]. On the other hand, in models of gastric mucosal damage [31,72], *Spirulina* or its active ingredients was effectively protective via antioxidant and anti-inflammatory mechanisms. Taken together, these results corroborate the findings of the present study.

Our results here demonstrated that the HA extract of *Spirulina platensis* was an effective alternative to SSZ treatment in mitigating the inflammatory actions induced by DSS administration via modulation of inflammation and tissue damage, which was positively reflected in preservation of animals’ body weight and lower DAI scores, unlike the chloroform extracts, which showed modest effects on preservation of body weight and minimal effects on DAI scores. Importantly, HA extraction solvent composition (40:60 water to alcohol) has been previously reported to be optimum for lipid-soluble ingredient extraction in microalgal preparations [73]. In addition, a recent study showed that HA extraction of previously extracted *Spirulina platensis* biomass using a different composition (80% ethanol) HA mixture produced the highest carotenoid yield [74]. The results of our study showed the presence of carotenoids in the HA extracts, as evidenced by chromatographic analysis findings. The better protective effects displayed by the HA extract in the current study (compared to the chloroform extract) might be attributable to its content of antioxidant and anti-inflammatory ingredients, including carotenoids and possibly other ingredients. A strong body of evidence showing the positive effects of *Spirulina* species on body retinoid composition and antioxidant activity [66,75,76] supports our hypothesis that antioxidant ingredients, presumably carotenoids, are at least in part critical for the protective effects of the HA extract of *Spirulina* against experimental colitis. Moreover, previous studies have shown that HA extracts can generally be richer in polyphenols and other bioactive compounds than chloroform extracts [77], and that variation in solvent composition results in variation in the active constituents of cyanobacteria extracts [78].

The limitations of the current study include the use of limited numbers of experimental animals (six animals per group). In addition, this work was carried out as a preventive study, where different treatments were commenced in the first day of experiment concurrently with DSS. Moreover, although the protection imparted by *Spirulina platensis* was compared to a widely-accepted clinically-used treatment in IBD, SSZ, one important limitation of the current study is the lack of direct evidence to show the role of carotenoids, and possibly other ingredients, in the protective effects displayed by the HA extracts. However, clinical and experimental evidence by others has highlighted the importance of carotenoids in the management of gastrointestinal inflammatory conditions, including UC [19,21,28,29,61,68].

The results of the current experimental study, despite its importance, cannot be directly extrapolated to clinical applications in UC patients. However, given the popularity of *Spirulina* as a functional food, and its expected safety, the results of the current study and previous work in the literature warrant the design and execution of clinical trials to investigate the possible protection by this blue-green alga in human UC and other inflammatory pathologies.

## 5. Conclusions

The results of the present study introduced the HA extract of *Spirulina platensis* as an effective treatment of UC in a well-established experimental model. The mechanisms involved in such protection were shown to be via modulation of local and systemic inflammation, which does not exclude the possible contribution of other mechanisms, notably its antioxidant effects.

## Figures and Tables

**Table 1:** Chemical nature of the compounds identified in *Spirulina platensis* hydroalcoholic extract using gas chromatography–mass spectrometry.

Chemical Nature	Total Number
Amino acids	4
Secondary metabolites	3
Organic acids	7
Terpenes	1
Fatty acids	5
Others	4

**Table 2:** Compounds identified in *Spirulina platensis* hydroalcoholic extract using gas chromatography–mass spectrometry.

	Compound Name	RT	TMS Derivative	KEGG ID/Chem ID	Qualification Ions
**1**	Methylamine	7.973	2 TMS	C00218	175, 160
**2**	4-Hydroxybutanoic acid	8.451	2 TMS	C01089	248, 247
**3**	L-Alanine	9.474	1 TMS	C00041	161, 146
**4**	Ethylene glycol	10.296	2 TMS	C01380	206, 191
**5**	Dimethylglycine	10.456	1 TMS	C01026	175, 160
**6**	L-Valine	11.705	2 TMS	C00183	261, 246
**7**	Lactic acid	11.806	2 TMS	C00186	234, 219
**8**	Acetic acid	12.142	2 TMS	C00033	220, 205
**9**	Glycerol	12.754	3 TMS	C00116	308, 293
**10**	Succinic acid	14.147	2 TMS	C00042	262, 247
**11**	Glyceric acid	14.558	3 TMS	C00258	322, 307
**12**	Uracil	14.801	2 TMS	C00106	256, 255
**13**	L-Isoleucine	15.799	2 TMS	C00407	275, 260
**14**	Phenylacetic acid	16.059	1 TMS	C07086	208, 193
**15**	Butanoic acid	16.881	3 TMS	C00246	336, 321
**16**	Erythronic acid	18.811	4 TMS	2781043	424, 409
**17**	Pyrogallol	19.993	3 TMS	C01108	342, 327
**18**	p-Hydroxyphenylacetic acid	20.539	2 TMS	C00642	296, 281
**19**	Myristic acid	22.82	1 TMS	C06424	300, 385
**20**	Palmitelaidic acid	24.666	1 TMS	5282745	326, 311
**21**	Palmitic acid	24.892	1 TMS	C00249	328, 313
**22**	10-Heptadecenoic acid	25.689	1 TMS	C00249	340, 325
**23**	Phytol	26.167	1 TMS	C01389	368, 353
**24**	Linoleic acid	26.822	1 TMS	C01595	352, 337

**Table 3:** Effect of dextran sulfate sodium (DSS)-induced ulcerative colitis (UC) on body weights of rats and its modulation by various treatments.^1^

Day	Positive (DSS)	Normal	Standard (SSZ)	Chloroform (Low)	Chloroform (High)	HA (Low)	HA (High)
**1**	220.33 ± 8.64	224.83 ± 10.26	217.50 ± 4.90	226.41 ± 14.67	231.52 ± 7.25	229.83 ± 8.62	226.54 ± 6.88
**8**	198.50 ± 7.69 ^c^	230.25 ± 7.08 ^a^	206.67 ± 7.03	208.00 ± 8.92 ^b^	220.13 ± 6.17 ^a^	220.83 ± 6.56 ^a^	228.83 ± 3.94 ^a^
**15**	174.00 ± 5.71 ^cd^	231.17 ± 5.83 ^a^	211.83 ± 8.42 ^a^	197.33 ± 8.18 ^ab^	215.33 ± 5.12 ^a^	222.50 ± 6.49 ^a^	229.96 ± 7.81 ^a^

^1^ Animals were weighed (g) before the start of the experiment (Day 1) and on Days 8 and 15. The data (mean ± SD) obtained from various groups were statistically analyzed using two-way ANOVA followed by Tukey’s multiple comparison test. a: *p* < 0.001 compared to positive (DSS) group at the same day; b: *p* < 0.05; c: *p* < 0.001 compared to Day 1 in the same group; d: *p* < 0.001 compared to Day 8 in the same group. Low and high are 100 and 200 mg/kg of *Spirulina platensis* extracts. HA: Hydroalcoholic; SSZ: Sulfasalazine.

**Table 4:** Effect of dextran sulfate sodium (DSS)-induced ulcerative colitis (UC) and various treatments on blood inflammatory markers.

Parameter	Positive (DSS)	Normal Control	Standard (SSZ)	Chloroform (Low)	Chloroform (High)	HA (Low)	HA (High)
**TNF-α (ng/mL)**	2.2 ± 0.118 ^#^	1.33 ± 0.124	1.14 ± 0.103	1.83 ± 0.115 *^abc^	1.74 ± 0.154 *^abc^	1.41 ± 0.136	1.16 ± 0.119 ^b^
**IL-6 (ng/mL)**	7.09 ± 0.187 ^#^	1.24 ± 0.116	1.79 ± 0.127 *	5.20 ± 0.124 *^ab^	3.11 ± 0.153 *^ab^	2.07 ± 0.138 *	1.68 ± 0.104 *^b^
**ESR (mm/h)**	8.61 ± 0.93	2.71 ± 0.55 ^d^	5.04 ± 0.53 ^d^	5.79 ± 1.76 ^e^	5.55 ± 1.86 ^fg^	6.08 ± 1.47 ^eh^	3.72 ± 1.36 ^dg^

TNF-α: tumor necrosis factor-α, IL-6: interleukin-6, ESR: Erythrocyte sedimentation rate. Normal control: vehicle-treated normal animals, DSS: dextran sulfate sodium positive control, SSZ: animals received DSS + sulfasalazine, Chloroform (low): animals received DSS + small dose of chloroform extract of *Spirulina platensis*, Chloroform (high): animals received DSS + high dose of chloroform extract of *Spirulina platensis*, HA (low): animals received DSS + small dose of hydroalcoholic extract of *Spirulina platensis*, HA (high): animals received DSS + high dose of hydroalcoholic extract of *Spirulina platensis*. Data (mean ± SD, n=6) were analyzed by one-way ANOVA followed by Tukey’s multiple comparisons test. # *p* < 0.001 compared with all other groups; * *p* < 0.001 compared with control group; a *p* < 0.01 compared with SSZ group; b *p* < 0.05 compared to low dose HA extract; c *p* < 0.05 compared to high dose HA extract; d *p* < 0.001, e *p* < 0.05, f *p* < 0.01 compared to DSS group; g *p* < 0.01, h *p* < 0.05 compared to the control group.

**Table 5:** Effect of dextran sulfate sodium (DSS)-induced ulcerative colitis (UC) and various treatments on tissue myeloperoxidase (MPO) activity.

Parameter	Positive (DSS)	Normal Control	Standard (SSZ)	Chloroform (Low)	Chloroform (High)	HA (Low)	HA (High)
**MPO (ng/mg protein)**	40.50 ± 2.46 ^a^	25.23 ± 2.37	20.48 ± 2.68 ^#^	37.18 ± 2.62 ^##@@^	33.07 ± 2.88 ^##@@^	26.53 ± 1.92 ^@b^	24.00 ± 1.98 ^b^

Normal control: vehicle-treated normal animals, DSS: dextran sulfate sodium positive control, SSZ: animals received DSS + sulfasalazine, Chloroform (low): animals received DSS + small dose of chloroform extract of *Spirulina platensis*, Chloroform (high): animals received DSS + high dose of chloroform extract of *Spirulina platensis*, HA (low): animals received DSS + small dose of hydroalcoholic extract of *Spirulina platensis*, HA (high): animals received DSS + high dose of hydroalcoholic extract of *Spirulina platensis*. Data (mean ± SD, n = 6) were analyzed by one-way ANOVA followed by Tukey’s multiple comparisons test. a *p* < 0.001 compared with all other groups; # *p* < 0.05 and ## *p* < 0.001 compared to control group. @ *p* < 0.01 and @@ *p* < 0.001 compared to SSZ group; b *p* < 0.001 compared to the chloroform extract doses.
